# Complete mitochondrial genome of the dark mealworm *Tenebrio obscurus* Fabricius (Insecta: Coleoptera: Tenebrionidae)

**DOI:** 10.1080/23802359.2018.1437800

**Published:** 2018-02-09

**Authors:** Yu Bai, Can Li, Min Yang, Shen Liang

**Affiliations:** a Guizhou Provincial Key Laboratory for Rare Animal and Economic Insects of the Mountainous Region, Guiyang University, Guiyang, China;; b XD-Tech Co., Ltd, Guiyang, China;; c College of Mathematics and Information Science, Guiyang University, Guiyang, China

**Keywords:** *Tenebrio obscurus*, Tenebrionidae, resource insect, dark meal worm, mitochondrial genome

## Abstract

The dark meal worm *Tenehrio obscurus* (Fabricius) is frequently found as a pest of stored grain, but it is an important resource insect like the yellow meal worm (*Tenebrio molitor*), larvae of which has high-protein content. The mitochondrial genome of *T. obscurus* is a circular molecule of 15,771 bp (GenBank accession number MG739327), with A + T bias of 72.69%. It comprises 13 protein-coding, 22 tRNA and two rDNA genes. The protein-coding genes had typical ATN (Met) as the initiation codon and were terminated by the typical stop codon TAN, except for COX2, COX3, nadh5 and nadh4. The phylogenetic tree based on maximum-likelihood method showed that the phylogenetic position of *T. obscurus* is closely related to *T. molitor*.

The dark meal worm (*Tenehrio obscurus* (Fabricius)) is a beetle frequently found as a pest of stored grain (Dillon and Dillon 1961), larvae of which is an important animal feed additive like the yellow meal worm (*Tenebrio molitor*) because of its high-protein content (Yi et al. 2010; Liu and Wang 2014). *Tenehrio obscurus* is very closely related to *T. molitor* (Plohl and Ugarković 1994; Rees 2004). There are still some clear needs for information determined for the genetic background of this beetle in both nuclei and organelles, which has not yet been determined.

Sample of adult *T. obscurus* was collected from Guiyang City, Guizhou Province of China, on 21 June 2017, which has been maintained in our laboratory since March 2010 (GYU accession number 201003-010). Genomic DNA isolated from the worker was fragmented for 250 bp by Covaris M220 (Covaris, Inc., Woburn, MA), built up genomic library, and sequenced (pair-end 2 × 150 bp) using Illumina Hiseq X Ten (Illumina, San Diego, CA). We obtained approximately 10,672 Mb of raw data and 10,005 Mb of clean data. Genome was *de novo* assembled by the SOAPdenovo v2.04 software (http://soap.genomics.org.cn/soapdenovo.html) (Luo et al. 2012).

The *T. obscurus* mitochondrial genome forms a 15,771 bp closed loop, which is biased towards A + T nucleotides, accounting for A (43.31%), T (29.38%), C (17.19%), and G (10.13%). AT-skew and GC-skew of the major strand of mt genome were approximately 0.1917 and −0.2896, respectively, using the formula proposed by Perna and Kocher (1995). The length of A + T-rich region of mt genome was 1177 bp, which located between the 12S rRNA and tRNAIle.

The complete mt genome of *T. obscurus* contains 13 protein-coding genes (PCGs), 22 tRNA, and two rRNA. The 13 PCGs of *T. obscurus* used standard ATN (Met) start codon, including four ATAs (COX1, COX2, nadh5, and nadh1), four ATTs (nadh2, ATP8, nadh3, and nadh6), five ATGs (ATP6, COX3, nadh4, nadh4L, and cytB). COX1, nadh2, ATP8, ATP6, nadh4L, nadh6 and cytB genes had the common stop codon TAA; nadh3 and nadh1 had the common stop codon TAG; while COX2, COX3, nadh5 and nadh4 terminated with incomplete stop codon T completed by the addition of 3’ A residues to the mRNA. The 22 tRNA genes identified within the *T. obscurus* mtgenome were interspersed throughout the coding region and ranged from 59 to 69 bp in length. The size of lrRNA and srRNA genes of the *T. obscurus* mtgenome were 1256 and 762 bp long, respectively.

To validate the phylogenetic position of *T. obscurus*, 13 mitochondrial protein-coding genes of the complete mitochondrial DNA sequences from 19 closely related taxa, including 6 species from Tenebrionidae, constructed a maximum-likelihood tree using RAxML version 8.0.0 software (https://sco.h-its.org/exelixis/web/software/raxml/index.html) (Stamatakis 2014) (Figure 1). The phylogenetic position of *T. obscurus* was closely clustered with *T. molitor*, and *Ulomoides dermestoides*. In conclusion, the first complete mtDNA of *T. obscurus* is decoded in this study and provides essential and important DNA molecular data for further phylogenetic and evolutionary analysis for Tenebrionidae.

**Figure 1.**
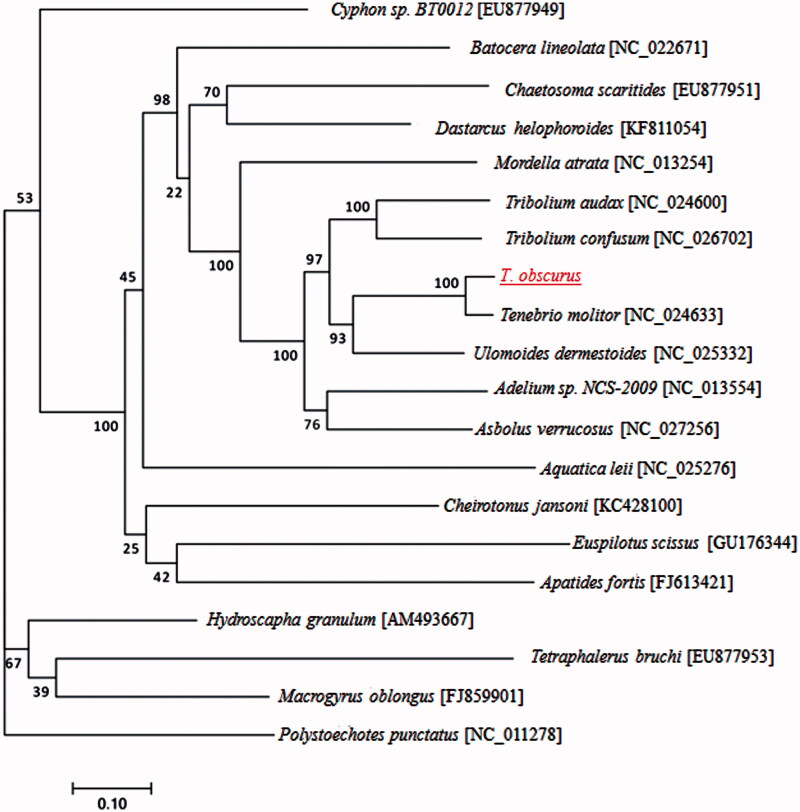
The maximum likelihood phylogenetic tree of *T. obscurus* and other beetles based on the nucleotide sequence of 13 mitochondrial protein-coding genes regions.
